# β-D-Glucoside utilization by *Mycoplasma mycoides *subsp. *mycoides *SC: possible involvement in the control of cytotoxicity towards bovine lung cells

**DOI:** 10.1186/1471-2180-7-31

**Published:** 2007-04-17

**Authors:** Edy M Vilei, Ivone Correia, M Helena Ferronha, Daniela F Bischof, Joachim Frey

**Affiliations:** 1 Institute of Veterinary Bacteriology, University of Bern, Länggass-Strasse 122, Postfach, CH-3001 Bern, Switzerland; 2 Laboratório Nacional de Investigação Veterinária, Departamento de Biologia Celular, Estrada de Benfica 701, P-1549-011 Lisbon, Portugal

## Abstract

**Background:**

Contagious bovine pleuropneumonia (CBPP) caused by *Mycoplasma mycoides *subsp. *mycoides *small-colony type (SC) is among the most serious threats for livestock producers in Africa. Glycerol metabolism-associated H_2_O_2 _production seems to play a crucial role in virulence of this mycoplasma. A wide number of attenuated strains of *M. mycoides *subsp. *mycoides *SC are currently used in Africa as live vaccines. Glycerol metabolism is not affected in these vaccine strains and therefore it does not seem to be the determinant of their attenuation. A non-synonymous single nucleotide polymorphism (SNP) in the *bgl *gene coding for the 6-phospho-β-glucosidase (Bgl) has been described recently. The SNP differentiates virulent African strains isolated from outbreaks with severe CBPP, which express the Bgl isoform Val_204_, from strains to be considered less virulent isolated from CBPP outbreaks with low mortality and vaccine strains, which express the Bgl isoform Ala_204_.

**Results:**

Strains of *M. mycoides *subsp. *mycoides *SC considered virulent and possessing the Bgl isoform Val_204_, but not strains with the Bgl isoform Ala_204_, do trigger elevated levels of damage to embryonic bovine lung (EBL) cells upon incubation with the disaccharides (i.e., β-D-glucosides) sucrose and lactose. However, strains expressing the Bgl isoform Val_204 _show a lower hydrolysing activity on the chromogenic substrate *p*-nitrophenyl-β-D-glucopyranoside (pNPbG) when compared to strains that possess the Bgl isoform Ala_204_. Defective activity of Bgl in *M. mycoides *subsp. *mycoides *SC does not lead to H_2_O_2 _production. Rather, the viability during addition of β-D-glucosides in medium-free buffers is higher for strains harbouring the Bgl isoform Val_204 _than for those with the isoform Ala_204_.

**Conclusion:**

Our results indicate that the studied SNP in the *bgl *gene is one possible cause of the difference in bacterial virulence among strains of *M. mycoides *subsp. *mycoides *SC. Bgl does not act as a direct virulence factor, but strains possessing the Bgl isoform Val_204 _with low hydrolysing activity are more prone to survive in environments that contain high levels of β-D-glucosides, thus contributing in some extent to mycoplasmaemia.

## Background

*Mycoplasma *species are known to represent the smallest self replicating organisms on earth, possessing the smallest genomes that contain basically fundamental functions to ensure autonomous life. Potential virulence genes are found in many mycoplasmas [1-9]. However, the genome of *Mycoplasma mycoides *subsp. *mycoides *small-colony type (SC), a *Mycoplasma *species causing one of most severe infectious animal diseases as defined by the World Organization for Animal Health and included in the list of diseases notifiable to the Office International des Epizooties (OIE), does not show classical virulence genes such as toxin, cytolysin and invasin genes [10]. *M. mycoides *subsp. *mycoides *SC causes contagious bovine pleuropneumonia (CBPP), the most serious cattle disease in Africa since the successful control of rinderpest [11]. In Europe, CBPP re-emerged at the end of the last century after being completely eradicated between 1967 and the 1980s [12], thus showing that CBPP is still a threat also to continents that have been able to eradicate the disease.

Virulence of *M. mycoides *subsp. *mycoides *SC seems to be determined by intrinsic functions such as: i) capsular polysaccharide (CPS) that seems to be involved in serum-resistance [13-17]; ii) lipoproteins that are expected to play a role as triggers in mechanisms of pathogenicity and in the release of pro-inflammatory cytokines [18-22]; iii) yet unknown but necessary adhesion factors that may play a central role in the intimate interactions of pathogenic mycoplasmas with mammalian cells for long periods thus triggering a cascade of signals which are transduced to the host cell and induce inflammation [23]; iv) repeating elements in variable membrane proteins of mycoplasmas that are suggested to increase the pathogen's ability to adhere to host cells and to evade the host immune response [24-26]; v) immunomodulating factors that can cause apoptosis of the mononuclear cells triggered by live *M. mycoides *subsp. *mycoides *SC or by a substance released by these mycoplasmas [27]; and vi) toxic metabolic side products such as H_2_O_2 _[28-31], which is translocated efficiently to the host cells where it can cause cell death [32].

Since no genetic tools for targeted mutagenesis are available for *M. mycoides *subsp. *mycoides *SC, analysis of virulence factors requires the use of various strains, including field strains from outbreaks of CBPP with various degrees of severity, strains from geographically different areas and live vaccine strains. Detailed genetic studies comparing *M. mycoides *subsp. *mycoides *SC isolates has provided evidence that deletion of *gtsC *and part of *gtsB *(two of three genes involved in glycerol transport) in the moderately virulent European strains correlated with a reduced ability to produce H_2_O_2 _compared to the highly virulent strains of the African/Australian cluster [31]. Vaccine strains of *M. mycoides *subsp. *mycoides *SC such as T1/44, T1/Sr50 and KH_3_J possess however an intact glycerol uptake system and metabolism [33,34], which do not distinguish them from virulent field strains by means of H_2_O_2 _production. Hence, other functions that determine virulence are expected in *M. mycoides *subsp. *mycoides *SC. For instance, these vaccine strains seem to produce lower amounts of CPS compared to field strains [16]. CPS seems to play a role in the capacity of persistence and dissemination of *M. mycoides *subsp. *mycoides *SC in the infected host [17]. However, the genetic locus of the CPS regulation still remains unknown.

We have recently identified by PCR amplification and restriction enzyme analysis (PCR-REA) a genetic difference in a structural gene which divides *M. mycoides *subsp. *mycoides *SC strains in two groups, one including virulent African field strains and the other comprising the T1-derived vaccine strains T1/44 and T1/Sr50, as well as European and Australian strains and the type strain PG1 [35]. This genetic difference was found in the *bgl *gene that codes for 6-phospho-β-glucosidase (Bgl). Bgl is an enzyme that is associated with the phosphoenolpyruvate-dependent sugar:phosphotransferase system (PEP-PTS), a multicomponent system involved in the simultaneous translocation and phosphorylation of sugars by bacteria from both gram-positive and gram-negative genera [36]. The PEP-PTS and Bgl are involved in incorporation and subsequent catabolism of β-D-glucosides (e.g., disaccharides).

The present work focuses on the characterization of Bgl and related disaccharide catabolism, and the possible contribution of sugar metabolism to control cytotoxicity of *M. mycoides *subsp. *mycoides *SC.

## Results

### Genetic variability of the *bgl *gene in *M. mycoides *subsp. *mycoides *SC

**Figure 1:**
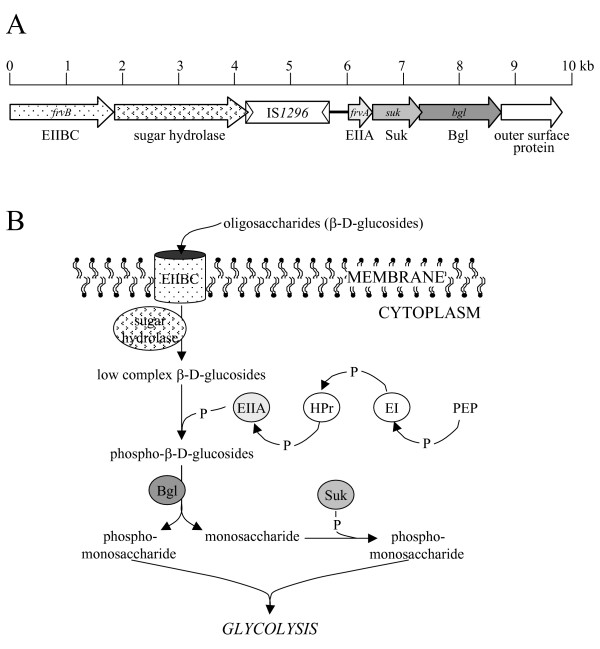
**Identification of the genes involved in oligosaccharide uptake and Bgl-dependent utilization in *M. mycoides *subsp. *mycoides *SC**. (A) Genetic map of the locus involved in the metabolism of β-D-glucosides. The open box indicates the IS element IS*1296 *and the large horizontal arrows indicate open reading frames found in the 9.8 kb DNA portion. (B) Model for the Bgl-dependent metabolism of β-D-glucosides in *M. mycoides *subsp. *mycoides *SC. Oligosaccharides are incorporated into the mycoplasma through the protein EIIBC. Once in the cytoplasm, sugar hydrolase may split complex β-D-glucosides into less complex β-D-glucosides (e.g., monosaccharides and disaccharides). Phosphorylation of these intracellular sugar molecules is preceded by the transfer of a phosphoryl group (P) from phosphoenolpyruvate (PEP) to EIIA in a pathway also involving enzyme I (EI) and the phosphoryl carrier protein (HPr). Then, Bgl hydrolyzes β-glycosidic linkages in the phospho-β-D-glucosides and residual monosaccharides are phosphorylated by the sugar kinase (Suk) before entering glycolysis.

The 9.8 kb genomic region containing the genes involved in disaccharide uptake and metabolism in *M. mycoides *subsp *. mycoides *SC type strain PG1, whose genome sequence was published by Westberg *et al*. [10], was considered in this study. Figure 1 shows the expected roles of EIIBC, sugar hydrolase, EIIA, Bgl and Suk in the disaccharide transport system and sugar metabolism. The *bgl *gene from ten selected strains of *M. mycoides *subsp *. mycoides *SC (Table 1) was sequenced with primers listed in Table 2. The sequences from the African field strains Afadé, 8740 and 91130 were identical, although these strains were isolated in geographically distant locations in an interval of as much as 23 years. The *bgl *sequences obtained from the vaccine strains T1/44 and T1/Sr50, as well as from type strain PG1, two European and two Australian strains presented a single nucleotide polymorphism (SNP) when compared to those of the African field strains. The non-synonymous SNP involves an amino acid change at position 204 of the Bgl protein sequence reported recently [35]. The group of the three African field strains possesses the Bgl isoform Val_204_, while the vaccine strains, the European and Australian outbreak strains and PG1 possess the Bgl isoform Ala_204_.

### Carbohydrate utilization of *M. mycoides *subsp. *mycoides *SC

The biochemical reactions of all strains tested indicated that *M. mycoides *subsp. *mycoides *SC can use the monosaccharides glucose, fructose and galactose, and the disaccharides sucrose and lactose. In fact, after incubation of mycoplasma suspensions in serum-free media with high amounts of either substrate in the presence of the indicator bromothymol blue for 6 days, all reactions turned light green to bright yellow (depending on the substrate) in colour, indicating production of CO_2 _and therefore carbohydrate uptake and utilization. Daily examination of the reactions revealed that there were no significant differences in the substrate utilization rates among the 5 strains tested (not shown).

### *M. mycoides *subsp. *mycoides *SC induction of cell damage in the presence of sugars

**Figure 2:**
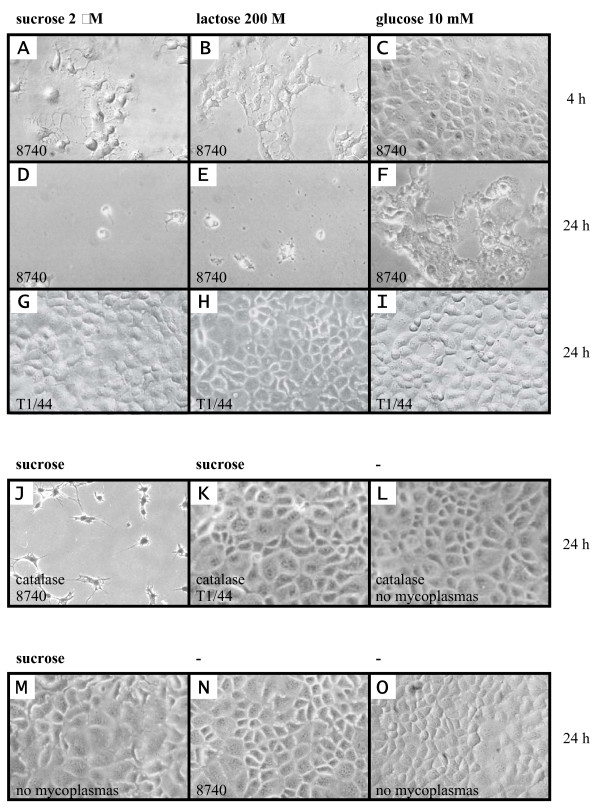
**Effect of sugars on cytotoxicity of *M. mycoides *subsp. *mycoides *SC on bovine lung cells in vitro**. Representative photomicrographs (320 ×) of EBL cell morphology upon mycoplasma infection in the presence of sugars. Pictures were taken after a 4 h incubation with the African field strain 8740 in the presence of sucrose (2 μM, panel A), lactose (200 μM, panel B) or the monosaccharide glucose (10 mM, panel C) and again after 24 h (panels D-F). After 24 h, pictures of EBL cell monolayers incubated with the less virulent vaccine strain T1/44 in the presence of these three sugars were also taken (panels G-I). Catalase (160 U/ml) was added to the EBL cells, which were then incubated for 24 h with strain 8740 and sucrose (panel J), or with strain T1/44 and sucrose (panel K). Controls performed to exclude eventual toxic effects of catalase alone (panel L), sugars alone (e.g., sucrose, panel M) or of strain 8740 without sugars (panel N) are also shown. Panel O shows EBL cells that were not treated at all.

The effect of *M. mycoides *subsp. *mycoides *SC on embryonic bovine lung (EBL) cells in the presence and absence of disaccharides was studied. Figure 2 shows the cell damage mediated by strain 8740, chosen as representative of *M. mycoides *subsp. *mycoides *SC strains with the Bgl isoform Val_204_, and of the vaccine strain T1/44, as representative for strains with the Bgl isoform Ala_204_. Cell damage was expressed by morphological changes mainly characterized by cell retraction, which was evident in monolayers of EBL cells inoculated with strain 8740 in the presence of sucrose or lactose but not in EBL cells inoculated with vaccine strain T1/44, with or without these disaccharides. The relative cell damage as a measure for cytotoxicity increased with increasing concentrations of the disaccharides (not shown). Morphological changes were already noticeable 3 h post-inoculation with strain 8740 in the presence of low concentrations of sucrose and lactose. At 4 h, those changes became easily observable (Figure 2, panels A and B) and were more evident at 8 h post-inoculation (not shown). Mycoplasmas incubated in the presence of monosaccharides for 4 h had no cytotoxic effects towards EBL cells (Figure 2, panel C).

After 24 h, cell monolayers infected with strain 8740 in the presence of sucrose and lactose had almost completely lost confluence and numerous detached rounded cells were observed (Figure 2, panels D and E). Morphological cell changes after 24 h were also seen with strain 8740 when incubated with glucose, fructose or galactose, but the degree of cell damage mediated by these monosaccharides was significantly lower when compared to that of the disaccharides (Figure 2, panel F). Strain T1/44 did not induce cell damage, either in the presence or absence of the five tested sugars (Figure 2, panels G, H and I). The same was observed with the European strains L2 and B345/93 (not shown).

The cytotoxic effect observed upon incubation with strain 8740 in the presence of the disaccharides could not be blocked when the cell monolayers were pre-treated with catalase at a concentration of 160 U/ml. Under these conditions, the EBL cells showed dramatic morphological changes 24 h post infection as in the absence of catalase (Figure 2, panel J). Strain T1/44 in the presence of disaccharides and catalase (Figure 2, panel K) and catalase alone (Figure 2, panel L) did not induce cell damage. Moreover, no morphological changes were detected when EBL cell monolayers were incubated either with sugars alone, with strain 8740 alone or with axenic medium (Figure 2, panels M, N and O).

### pNPbG hydrolysis of *M. mycoides *subsp. *mycoides *SC grown on solid medium

Hydrolysis of *p*-nitrophenyl-β-D-glucopyranoside (pNPbG; colourless) to give *p*-nitrophenol (yellow) was determined qualitatively to assess 6-phospho-β-glucosidase activity by flooding cultures of *M. mycoides *subsp *. mycoides *SC on nutrient agar plates with pNPbG solution. After 1 h, no colour change was seen with the African *M. mycoides *subsp *. mycoides *SC field strains tested (Afadé, 8740 and 91130), indicating no detectable hydrolytic activity of Bgl isoform Val_204_. In contrast, individual colonies of the other strains tested, i.e., the vaccine strains T1/44 and T1/Sr50, as well as the European strains B345/93 and L2 and the Australian strains Gladysdale and DVZ, which express the Bgl isoform Ala_204_, were coloured yellow, indicating hydrolysis of pNPbG (Table 1). It has to be noted that after prolonged incubation, the three African field strains also showed some hydrolysis of pNPbG, as revealed by a light yellow coloration in areas of confluent growth.

### Production of H_2_O_2 _by *M. mycoides *subsp. *mycoides *SC

Production of H_2_O_2 _after the addition of mono- and disaccharides to suspensions of *M. mycoides *subsp *. mycoides *SC strain 8740 and the vaccine strain T1/44, chosen as representative strains in this study, was measured. Addition of physiological concentrations of the different sugars to the two mycoplasmas tested resulted in a weak release of H_2_O_2 _into the incubation buffer after 1 h. The released H_2_O_2 _levels measured in the suspensions were at concentrations below or equal to 0.5 μg/ml, the detection level of the assay. In contrast, the addition of 100 μM glycerol to the mycoplasmas instantly resulted in a strong release of H_2_O_2 _into the incubation buffer, reaching 8.75 μg/ml by strain 8740, and 7.5–7.75 μg/ml by vaccine strain T1/44 after 1 h. All H_2_O_2 _concentrations measured were retained up to 2 h and showed no significant differences between the two strains.

### Viability of *M. mycoides *subsp. *mycoides *SC upon incubation with different sugars and growth analysis of surviving cells

Starvation in incubation buffer alone, or in incubation buffer supplemented with 5 mM glucose or 5 μM sucrose affected the viability of *M. mycoides *subsp. *mycoides *SC strains 8740 and T1/44. Over the first 4 h of starvation, there was an overall loss of 7–39% of viable mycoplasma cells. After 18 h of starvation, the number of CFU for strain T1/44 declined sharply to a rate of < 1% of the original in the three assays (loss of > 99%), while that for strain 8740 was reduced to < 1% in incubation buffer alone and in the presence of glucose, and to approximately 12% in the presence of sucrose (Table 3).

The estimated quantities of mycoplasma cells of strains 8740 and T1/44 grown for 2 days after a preceding impregnation phase of 18 h with sucrose or glucose were determined in the TaqMan real-time PCR with primers and probe listed in Table 2. The Ct values shown in Table 4 are mean values of two or more replicate experiments (*P *< 0.0001; ANOVA: single factor analysis). Results showed that pre-treatment with incubation buffer in the absence of sugars for 18 h completely blocked the growth of both strains. Also pre-incubation of the two strains of *M. mycoides *subsp. *mycoides *SC with 5 mM glucose in incubation buffer affected dramatically their growth rate and the amounts of mycoplasmas grown in the 2-day culture were below 4% when compared to those of non-treated mycoplasmas (Table 4). Pre-incubation with 5 μM sucrose blocked the subsequent growth of strain T1/44 (≈1%), while strain 8740 retained a growth of approximately 15% of the rate without treatment (Table 4).

## Discussion

A single genetic difference between African field strains of *M. mycoides *subsp. *mycoides *SC and T1-derived vaccine strains was reported recently in the genomic region harbouring the genes *frvA*, *suk *and *bgl *[35]. This genetic difference is an SNP in the *bgl *gene coding for the 6-phospho-β-glucosidase (Bgl). Here we report the correlation of the two isoforms of Bgl to the cytotoxic potential of *M. mycoides *subsp. *mycoides *SC.

In cytotoxicity studies where we examined the effect of *M. mycoides *subsp. *mycoides *SC grown in the presence of certain mono- and disaccharides towards cultivated embryonic bovine lung (EBL) cells, the group of strains expressing the Bgl isoform Val_204 _showed high cytotoxicity in the presence of β-D-glucosides. This group contained principally highly virulent African field strains such as strains Afadé and 8740 [37-39]. In contrast, strains with the Bgl isoform Ala_204 _showed virtually no cytotoxicity in the presence of the two disaccharides sucrose or lactose. Most of the strains in this group are considered to be less virulent, in particular the vaccine strains T1/44 and T1/Sr50 used in this study [12,40]. Other strains in this group are the type strain PG1 that is not considered to be virulent (probably due to a high number of in vitro passages [41]), the European strains from outbreaks of 1980–2000, which primarily caused epidemics with low mortality [42] and of which a characteristic strain was shown experimentally to be of low virulence [37,38], and the Australian strains whose virulence in relation to the former strains has not been described. Other disaccharides such as the α-D-glucosides maltose and trehalose were not tested, as it has been shown that *M. mycoides *subsp. *mycoides *SC are unable to metabolize them [43-45]. Qualitative determination of pNPbG hydrolysis by *M. mycoides *subsp. *mycoides *SC grown on solid medium indicated that strains considered virulent and expressing the Bgl isoform Val_204 _have a reduced 6-phospho-β-glucosidase activity. It is worthwhile noting at this point, that the pattern and rates of disaccharide utilization were similar among the *M. mycoides *subsp. *mycoides *SC strains tested and thus there were no differences in substrate uptake that could account for different rates of pNPbG hydrolysis, which could thus be appropriately referred to as Bgl activity. However, it has to be considered that the SNP was found in the *bgl *gene, while no genetic differences were found in the genes *frvA *and *suk*, but it is possible that differences in *frvB *or in the sugar hydrolase gene are also present and could account for different pNPbG hydrolysis patterns as well. Other sugar metabolism genes are likely to be involved in the flow of β-D-glucosides into glycolysis, thus contributing to the similar pattern of disaccharide utilization among strains. In this regard, there are a number of other PTS enzymes or sugar metabolism proteins that are annotated in the genome of *M. mycoides *subsp *. mycoides *SC [10] and which may also be involved in β-D-glucoside metabolism.

Glucosides like disaccharides are among the most important and most common carbon and energy sources for bacteria. Disaccharides are accumulated as phosphorylated derivatives by the translocation-phosphorylation mechanism PEP-PTS and are cleaved by intracellular substrate-specific phospho-glycosylhydrolases such as Bgl [36]. Specifically, Bgl hydrolyzes the β-glycosidic linkage between the anomeric carbon and glycosidic oxygen of intracellular phospho-β-D-glucosides. The residual monosaccharides obtained by this enzymatic reaction are then phosphorylated before being metabolized during glycolysis or undergo other metabolic reactions such as sugar oxidation. In mycoplasmas, oxidation of sugars together with oxidation of organic acids or glycerol lead to production of H_2_O_2 _[46]. Glycerol-dependent H_2_O_2 _production was shown to be a virulence determinant in *M. mycoides *subsp. *mycoides *SC and attenuated strains were found to have a defect in glycerol-related H_2_O_2 _generation [31,32]. In contrast, the amount of H_2_O_2 _produced by mycoplasmas grown in the presence of sugars is approximately 20 times less if compared to that produced in the presence of glycerol [47]. The low production of H_2_O_2 _by *M. mycoides *subsp. *mycoides *SC upon incubation with sugars was confirmed in our work and, moreover, in our cytotoxicity studies we could demonstrate that sugar-mediated H_2_O_2 _production by this mycoplasma does not contribute to induction of cell damage in the host, as addition of catalase, which behaves as a catalyst for the conversion of H_2_O_2 _into water and oxygen, could not block the cytotoxic effect.

Our study indicated that cytotoxicity of *M. mycoides *subsp. *mycoides *SC is related to defective Bgl activity. We thus assumed that β-D-glucosides could mediate in some extent repression of certain virulence factors of *M. mycoides *subsp. *mycoides *SC, as already reported for other pathogens [48,49]. This mechanism is known as carbon catabolite repression (CCR). In most bacteria, the enzymes involved in sugar transport and phosphorylation are known to play an essential role in signal generation leading through different transduction mechanisms to CCR that may mediate downregulation of virulence genes [50]. Viability tests and growth analysis of mycoplasma cells that have come through preceding impregnation with sugars in medium-free buffers indicated however that Bgl activity was rather involved in the control of growth of *M. mycoides *subsp. *mycoides *SC. In fact, the African field strains that possess the defective Bgl isoform Val_204 _were found to be more able than vaccine strains, which express the Bgl isoform Ala_204_, to preserve viable mycoplasma cells in the presence of β-D-glucosides such as sucrose or lactose, i.e., strains that seem to be particularly virulent are capable to subsist in some degree in environments that contain β-D-glucosides, while strains that have an attenuated virulence are not prone to β-D-glucosides and may succumb. Bacterial growth inhibition by elevated sugar metabolism was already described for *Escherichia coli *[51]. Two different mechanisms for this cessation of growth and substantial loss of viability were distinguished. One mechanism involved cell killing and the other mechanism resulted in growth inhibition without cell death. Cell killing was related to increased flux through glycolysis and consequent overproduction of the toxic metabolite methylglyoxal. Inhibition of growth resulted from excessive accumulation of organophosphates in the cell or depletion of inorganic phosphate pools [51]. It is possible that one or both of these "self-inflicted injury" mechanisms for growth inhibition also occurs in strains of *M. mycoides *subsp. *mycoides *SC considered of low virulence and possessing the active Bgl isoform Ala_204 _upon elevated utilization of β-D-glucosides. On the other hand, strains possessing the defective Bgl isoform Val_204 _do not metabolize β-D-glucosides in the same "self-inflicting" way and may rather use the latter in a different molecular mechanism of pathogenicity that enables them to exert cytotoxicity on host cells.

Almost no EBL cell damage was observed upon incubation with mycoplasmas in the presence of monosaccharides. Minor cell damage was observed only with strains 8740 or Afadé after a prolonged incubation of 24 h with glucose, fructose or galactose, while T1/44 was completely non-cytotoxic upon monosaccharide incubation. This indicated the presence of additional determinant(s) of cytotoxicity other than defective Bgl activity and glycerol metabolism in *M. mycoides *subsp. *mycoides *SC. A possible explanation why significant cytotoxicity in our experiments was only seen in the presence of some strains with sucrose and lactose, and not with monosaccharides, can reside in the fact that *M. mycoides *subsp. *mycoides *SC may be sensitive to high levels of monosaccharides. Note that the determined saturation constants (*K*_m_) for glucose and fructose in *M. mycoides *subsp. *mycoides *SC are < 5 μM [44,52], while we have applied calculated physiological concentrations for monosaccharides which were significantly (up to > 3 log) higher than the *K*_m _values. It is possible that such high carbohydrate concentrations may determine a high sugar metabolism rate and, consequently, a high production of toxic metabolites. The latter, in turn, may induce cessation of growth of all *M. mycoides *subsp. *mycoides *SC isolates, similarly to what observed upon starvation of less cytotoxic strains with β-D-glucosides.

As gene inactivation by homologous recombination [53] is currently impossible to achieve in *M. mycoides *subsp. *mycoides *SC (C. Janis & A. Blanchard, personal communication), no *bgl *inactivation could be assessed in the vaccine strain T1/44 in order to directly verify if the difference in the *bgl *gene is related to modulation of cytotoxicity of *M. mycoides *subsp. *mycoides *SC or not. However, there is evidence that the detected difference in the Bgl isoforms Ala_204 _and Val_204 _is close to (hence can influence) the catalytic site of the 6-phospho-β-glucosidase, as evidenced in an in silico three-dimensional (3D) localization of amino acid 204 of Bgl (see additional files 1 and 2). As no 3D structures are available for Bgl of *M. mycoides *subsp. *mycoides *SC, comparison of the Bgl sequence from strain Afadé with available sequences from the NCBI site was performed with the Conserved Domain Database (CDD). Alignment with sequences belonging to the cluster of orthologous groups of proteins COG2723 (BglB: β-glucosidase/6-phospho-β-glucosidase/β-galactosidase) and to the protein domain family pfam00232 (glyco_hydro_1: glycosyl hydrolase family 1) was obtained (see additional file 1). Comparison of Bgl with related proteins that have known 3D structures revealed homologies with the chain A sequences of the β-glycosidase from *Sulfolobus solfataricus *(1GOW_A) [54], the β-glucosidase from *Zea mays *(1HXJ_A) [55], and the 6-phospho-β-galactosidase from *Lactococcus lactis *(1PBG_A) [56]. As 1PBG_A presented the highest homology with Bgl from *M. mycoides *subsp. *mycoides *SC among the three characterized proteins, the software Cn3D 4.1 from NCBI was used to visualize the 3D structure of 1PBG_A with the aim to envisage the structure of Bgl (see additional file 2). Protein 1PBG_A showed three Glu residues in the active site and a Val residue in its vicinity. These four amino acids are equivalent to the catalytic triad and the SNP-related Val residue (Val_204_) in the Bgl protein of African field strains of *M. mycoides *subsp. *mycoides *SC. Protein 1PBG_A is an active enzyme in *L. lactis *[57,58] but we do not have any information as to if its Val_185 _is essential or not for enzymatic activity. It has to be noted at this point, that a transition between Ala and Val not involving the catalytic site has been already reported to be implicated in modulation of enzyme activities [59-61]. Such amino acid substitutions may modify the steric properties that are associated with the enzymatic activities by prompting conformational changes near the active site. These conformational changes may arise from the fact that Ala and Val have a different side-chain volume and therefore a transition Ala to Val can confer a different secondary structure to the enzyme [62,63].

## Conclusion

To cause disease or to be virulent, *M.mycoides *subsp. *mycoides *SC possesses multiple molecular mechanisms of pathogenicity [64]. These mechanisms permit mycoplasma to adhere to specific host tissue, to evade the host's immune defence, to enable persistence and dissemination in the infected animal, to exert a cytotoxic effect, and to cause inflammation and disease symptoms. The loss of any of these mechanisms can lead to attenuation or loss of virulence. Our observations of significant differences in cytotoxicity among Bgl variants of *M. mycoides *subsp. *mycoides *SC grown in the presence of different sugars indicate that the β-D-glucoside metabolism is involved in control of pathogenicity of *M. mycoides *subsp. *mycoides *SC. This mechanism is independent of H_2_O_2 _production, in contrast to glycerol metabolism [31,32]. Rather, cytotoxicity observed in the presence of β-D-glucosides seems to be due to the effect of defective 6-phospho-β-glucosidase activity on the containment of cell death. At this point, it is important to know that lungs have an effective innate defence system to counteract the airborne bacteria that are constantly inhaled. These organisms land in the thin layer of the liquid that overlays the cells lining the inside of the lungs. The lung cells produce natural antibiotic substances, such as polypeptides and glycoproteins, and secrete them into the airway surface liquid where they kill the invading bacteria [65-67]. Also, degradation of the saccharide moiety of glycoproteins generates sugars [68], which, acting synergistically with other agents, may contribute to provide a defence barrier to colonization and infection in the host's lungs. As sugars are able to affect viability of those bacteria that are able to metabolize them in large amounts [51], a defective 6-phospho-β-glucosidase activity in strains harbouring the Bgl isoform Val_204 _can contribute indirectly to their higher virulence, due to the fact that they possess a sort of "resistance" to elevated levels of certain sugars and may use them as a supplement for cytotoxicity on host cells.

## Methods

### Mycoplasma strains, growth conditions and DNA extraction

Strains of *M. mycoides *subsp. *mycoides *SC used in this study are listed in Table 1. Mycoplasmas were grown in a standard mycoplasma medium (Axcell Biotechnologies, St. Genis l'Argentière, France) for 3 days at 37°C to a density of 10^8^-10^9 ^cells/ml or on solid mycoplasma agar medium (Axcell Biotechnologies). Growth and handling of live *M. mycoides *subsp. *mycoides *SC were performed in a biological safety laboratory fulfilling the BSL-3 containment safety standards. Lysis of mycoplasmas with GES buffer (5 M guanidium thiocyanate, 100 mM EDTA, 0.5%*N*-lauroylsarcosine) and extraction of genomic DNA were performed as previously described [69].

### Sequencing strategies

To sequence the *bgl *gene of the strains listed in Table 1, polymerase chain reaction (PCR) was first performed with a DNA thermal cycler Gene Amp 9600 (Applied Biosystems, Foster City, CA, USA) in a 50-μl reaction mixture [50 mM Tris-HCl, pH 9.2, 1.75 mM MgCl_2_, 16 mM (NH_4_)_2_SO_4_, 350 μM of each dNTP] that contained approximately 50 ng of genomic template DNA, 300 nM of oligonucleotide primers 4000bp_6L and 4000bp_2R (Table 2), and 1.75 U of a mixture of *Taq *and *Pwo *DNA polymerases (Expand Long Template PCR System kit, Roche Diagnostics, Rotkreuz, Switzerland). The mixtures were subjected to 2-min denaturation at 94°C followed by 35 cycles of amplification with the parameters: 30s at 94°C, 30s at 48°C, and 3 min extension at 68°C. Amplicons were purified with the High pure PCR product purification kit (Roche Diagnostics).

DNA sequencing of the purified amplicons was performed with a DNA Sequenator AB 3100 genetic analyzer and the *Taq *dye deoxy terminator cycle sequencing kit (Applied Biosystems) by primer walking using internal *bgl *primers (Table 2). Assembling of DNA sequences and alignments of sequenced segments were done using the program Sequencher 4.6 (GeneCodes, Ann Arbor, MI, USA). Comparisons of DNA sequences and their deduced amino acid sequences with the EMBL/GenBank database were performed using the BLAST programs blastn, blastx and blastp [70].

### Mycoplasma preparation for the assays

Mycoplasma cultures were grown to the exponential phase, determined by counting the number of colony forming units (CFU) on solid medium and by evaluating 3-(4,5-dimethyltiazol-2-yl)-2,5-diphenyl-tetrazolium bromide reduction rate as previously described [71]. After centrifuging the cultures at 36,000 × *g *for 30 min, pellets were resuspended in fresh medium to 50% of the initial volume. Aliquots of 5 ml were stored at -80°C. The day before the assays, these cultures were thawed and incubated overnight at 37°C. They were then centrifuged as stated above and the pellets were washed once and resuspended with the aid of a 27-gauge needle in the appropriate buffer or medium (depending on the assay). The number of CFU was counted and adjusted to the desired densities (see below).

### Carbohydrate utilization tests

Conventional biochemical tests were performed to measure the utilization of glucose, fructose, galactose, sucrose and lactose in a serum-free medium by *M. mycoides *subsp. *mycoides *SC strains L2, Afadé, 8740, T1/44 and Gladysdale (Table 1). Sera or heat-inactivated sera were omitted to avoid possible saccharolytic activity by enzymes that can give fallacious results in sugar fermentation tests. The sterile laboratory-prepared basal medium containing bovine serum albumin, lipids, glycerol and penicillin G was prepared as described [43]. The basal medium was supplemented with bromothymol blue as indicator, which detects the presence of CO_2 _in the media. Either filter-sterilized carbohydrate solutions were added to the sterile basal medium (pH 7.6) to give final concentrations of 0.1%. The biochemical tests were inoculated with approximately 10^8 ^CFU/ml *M. mycoides *subsp. *mycoides *SC from the pellet preparations described above, incubated at 37°C in ambient air for 6 days and examined daily for reactions. Media turning yellow in colour indicated a positive carbohydrate utilization test.

### Bovine lung cell cultivation

Embryonic bovine lung (EBL) cells (ACC192; DSMZ, Braunschweig, Germany) were cultivated in Eagle's Minimum Essential Medium (MEM; Gibco Life Technologies, Gaithersburg, MD, USA) containing 2 mM L-glutamine supplemented with 4% foetal bovine serum (Gibco Life Technologies) and 50 μg/ml gentamicin. This medium was previously filtered by 0.1 μm filters (PALL Gelman Laboratory, Ann Arbor, MI, USA). To obtain a confluent monolayer, 5 × 10^4 ^freshly trypsinized cells were inoculated into each cavity of 24-well tissue culture plates (NUNC Brand Products, Rochester, NY, USA) and incubated for approximately 48 h at 37°C in an atmosphere of 5% CO_2_. The MycoFluor KIT (Molecular Probes, Eugene, OR, USA) was used as described by the manufacturer to ensure that cell cultures were free of mycoplasma contamination.

### Effect of *M. mycoides *subsp. *mycoides *SC on EBL cells in the presence of different sugars

To study the cellular effect of mycoplasma metabolism in the presence of different sugars, pellets of *M. mycoides *subsp. *mycoides *SC strains Afadé, 8740, T1/44, B345/93 and L2 (Table 1) were resuspended in MEM (without supplements and gentamicin) at a density of approximately 5 × 10^8 ^CFU/ml and then used to infect EBL cells.

Different final concentrations of the disaccharides sucrose (2, 5 and 10 μM) and lactose (50, 100 and 200 μM), and of the monosaccharides glucose (2, 5 and 10 mM), fructose (50, 100 and 200 μM) and galactose (50, 100 and 200 μM) were used. The sugar concentrations corresponded to the physiological levels in bovine serum and were computed as follows: the lactose concentration was derived from previous work [72,73] and ranges from 40–60 μM up to 230 μM in lactating cows; the concentration applied for glucose was derived from physiological levels measured in adult cattle (2.5–4.2 mM, up to 8.9 mM in veal calves; J. Blum, personal communication). As no data were available for concentrations of the other three sugars in cattle, we have considered as references their standard values in human blood (approximately 2 μM sucrose, 30–50 μM fructose and 50–100 μM galactose). Prior to infection of EBL cells, equal volumes (250 μl) of mycoplasma suspensions (≈1.25 × 10^8 ^CFU) and sugars (2 × final concentration) were incubated for 20 min at 37°C. Cell monolayers (≈2 × 10^5 ^cells) were washed twice with Hank's Balanced Salt Solutions (Gibco Life Technologies) and then incubated with the inocula (500 μl) for 24 h. The EBL cells were thus infected at a multiplicity of infection (MOI) of 550–700 mycoplasmas per cell. In control experiments, 160 U/ml catalase (Sigma-Aldrich Chemie GmbH, Buchs, Switzerland) was added to the cell monolayers prior to addition of the 500-μl inocula in order to block the effect of H_2_O_2 _produced by mycoplasmas. Other controls consisted of cell monolayers inoculated with one of the following: i) MEM; ii) mycoplasmas; iii) sugars; and iv) catalase. EBL cell morphology was monitored by phase contrast every hour for 8 hours and at 24 h using a Leica DMIL microscope. Each assay was performed in triplicate.

### Bgl activity assay

6-Phospho-β-glucosidase activity was determined qualitatively by measuring the hydrolysis of the chromogenic substrate *p*-nitrophenyl-β-D-glucopyranoside (pNPbG, Merck KGaA, Darmstadt, Germany) by intact mycoplasmas as already described for a similar substrate [45]. This is actually a coupled assay because degradation of β-glucosides by *M. mycoides *subsp. *mycoides *SC occurs by phosphorylation of the sugar during transport followed by hydrolysis of the glucosidic bond by the cytoplasmic Bgl to release *p*-nitrophenol [48].

Mycoplasma pellets from preparations described above were resuspended at a density of approximately 10^8^ CFU/ml in fresh standard mycoplasma medium and aliquots of 5 μl of 10-fold dilutions (6 log units, from non-diluted to 10^-5^) were spotted on solid mycoplasma agar medium. *M. mycoides *subsp. *mycoides *SC were grown for 3 days at 37°C and then colonies were flooded with 600 μl of 2 mM pNPbG dissolved in incubation buffer (67.6 mM HEPES, pH7.3, 140 mM NaCl, 7 mM MgCl_2_). Incubation was continued for another 3 h at 37°C. The reaction was blocked by adding 600 μl of 500 mM Na_2_CO_3_; this stopped the hydrolysis reaction and enhanced the yellow coloration of colonies. Colony coloration was examined using a NIKON SMZ 1500 with 40 × and 100 × final magnifications.

### Quantification of H_2_O_2 _production

To measure H_2_O_2 _production, pellets of strains 8740 and T1/44 of *M. mycoides *subsp. *mycoides *SC (Table 1) resuspended at a density of approximately 10^9^ CFU/ml in pre-warmed incubation buffer were incubated at 37°C for 1 h. To induce H_2_O_2 _production, final concentrations of the disaccharides sucrose (5 μM) and lactose (100 μM) and of the monosaccharides glucose (5 mM), fructose (50 μM) and galactose (100 μM) were added to the mycoplasma suspensions. The production of H_2_O_2 _was measured with the peroxide test (Merck KGaA) as previously described [31,32] at time zero and after 1- and 2-h incubation at 37°C in the presence of sugars. Each assay was performed in duplicate. The samples were then subjected to the mycoplasma viability and growth assay (see below). As a control for induction of H_2_O_2 _production, the two mycoplasmas were incubated with 100 μM final concentration of glycerol.

### Viability measurement and growth analysis of surviving cells

Viability measurement of strains 8740 and T1/44 upon 2-h treatment with incubation buffer alone, with 5 μM sucrose or with 5 mM glucose was performed by counting the CFU formed on solid medium after another 2 h (totally 4 h incubation) and another 16 h (totally 18 h) incubations at 37°C of the above H_2_O_2 _quantification assays. Aliquots of 10 μl of 10-fold serial dilutions were streaked with sterile loops on the solid medium in specific sectors of the plates, which were labelled with the corresponding dilution factors.

For measuring mycoplasma growth after pre-treatment with 5 μM sucrose or 5 mM glucose, we took 30-μl aliquots from the above H_2_O_2 _quantification assay – originally containing approximately 3 × 10^7 ^CFU of strains 8740 and T1/44 incubated with the corresponding sugars – and kept them at 37°C for another 16 h (totally 18 h incubation). As controls, 30 μl of cells in incubation buffer for the two mycoplasmas (≈3 × 10^7 ^CFU) were kept at 37°C for 18 h in the absence of sugars. Cells were resuspended in 1 ml of fresh standard mycoplasma medium and incubation at 37°C was continued for another 2 days. Other controls consisted of 60 μl from 3-day-old cultures (≈3 × 10^7^ CFU) that were not pre-warmed with incubation buffer but were immediately diluted with 1 ml of fresh mycoplasma medium and then grown for 2 days. Mycoplasmas were then harvested by centrifugation at 8,000 × g for 10 min, washed and resuspended in 1 ml sterile purified water, and boiled for 10 min. These lysates were finally used to quantitatively evaluate the amount of mycoplasmas by TaqMan real-time PCR, where a time-dependent signal increase was taken as a measure for growth efficacy of the mycoplasmas, revealing their viability. Reactions were performed using 2.5 μl of each lysate, a 900 nM concentration of each TaqMan primer (Table 2) and 300 nM TaqMan probe (Table 2) in a 25-μl volume. Primers and probe specific to the lipoprotein gene *lppQ *of *M. mycoides *subsp. *mycoides *SC were designed using the Primer Express software (Applied Biosystems). 6FAM reporter dye and MGB quencher were affixed on the 5' and 3' ends of the probe, respectively. PCR reactions were run on an ABI 7500 instrument (Applied Biosystems) using the following cycling parameters: after one step at 50°C for 2 min and at 95°C for 10 min, 40 cycles of denaturation at 95°C for 15s and extension at 60°C for 1 min were performed. Real-time fluorescence measurements were taken and the cycle number at which the fluorescent signal crossed the threshold for each sample was recorded (Ct value). Each assay was repeated at least twice.

A standard curve was produced by analyzing 10-fold dilutions of the *lppQ*-containing plasmid pJFFLP4811 [74] containing 0.7 to 7 × 10^10 ^genome equivalents (geq) in the TaqMan assay three times. It was linear over a range of 8 log units, with amounts ranging from 7 to 7 × 10^7 ^geq in 2.5 μl of analyzed samples, showing a correlation coefficient of 0.9987 (*r*^2 ^= 0.9975). The estimated quantities of mycoplasma cells were determined using the formula geq = 1.05 × 10^12 ^× e^-0.65 × Ct^, generated by the linear regression of standard curve [Ct values vs. log_10_(geq)].

### Nucleotide sequence accession numbers

The EMBL/GenBank accession numbers for the nucleotide sequences of the *bgl *genes from seven of the ten *M. mycoides *subsp. *mycoides *SC strains used in this study (Table 1) have been deposited under accession numbers AM114901–AM114907.

## Authors' contributions

EMV, MHF and JF participated in the discussions on the study design. EMV, IC and MHF participated in analysis and interpretation of the data. EMV and DFB performed sequence analysis, quantification of H_2_O_2 _production and viability measurement by TaqMan. IC and MHF carried out the experiments with EBL cells. EMV, MHF and JF participated in the writing of the manuscript. EMV was the principal author of the manuscript. All authors read and approved the final manuscript.

## Supplementary Material

Additional file 1 **Alignment of the Bgl amino acid sequence with those of related proteins**. The Bgl sequence from strain Afadé was aligned with the protein sequences COG2723, pfam00232, 1PBG_A, 1HXJ_A and 1GOW_A using the program DIALIGN 2, and the alignment was elaborated with Boxshade 3.21. The sequences for COG2723 (from *Enterococcus faecium*) and pfam00232 (from *Streptococcus pneumoniae*) were obtained with blastp and showed very high similarities with the Bgl sequence from *M. mycoides *subsp. *mycoides *SC. Black backgrounds indicate identical amino acids and grey backgrounds indicate similar residues. Asterisks (*) indicate the three Glu residues of the catalytic site and the circle (o) indicates the amino acid 204 in the Bgl sequence from *M. mycoides *subsp. *mycoides *SC. Click here for file

Additional file 2 **Three-dimensional model showing the secondary structures in the vicinity of the active site of 1PBG_A**. The "worm" model was obtained with the program Cn3D 4.1. The residues Glu_160_, Glu_320 _and Glu_375 _that comprise the catalytic site are shown in yellow. The Val_185 _(corresponding to Val_204 _in the Bgl of *M. mycoides *subsp. *mycoides *SC strain Afadé) is shown in green. All other residues are shown in pink. Note that Glu_320 _was not displayed by Cn3D 4.1, as Bgl amino acids 315–324 do not have a well-defined secondary structure, therefore its approximate location was introduced manually. Click here for file

## Figures and Tables

**Table 1:** *M. mycoides *subsp. *mycoides *SC strains used

Strain^a^	Origin	Year Isolated	Host	Bgl SNP^b^	Bgl activity^c^	Accession number or reference^d^
PG1	Unknown	1931	Cattle/type strain	Ala	ND	[10]
B345/93	Portugal	1993	Cattle	Ala	+	AM114901
L2	Italy	1993	Cattle/lung	Ala	+	AM114902
Afadé	Cameroon	1968	Cattle/lung	Val	-	[35]
8740	Cameroon	1987	Cattle	Val	-	AM114903
91130	Central African Republic	1991	Cattle	Val	-	AM114904
T1/44	Tanzania	1952	Cattle/vaccine strain	Ala	+	AM114905
T1/Sr50	Tanzania	1952	Cattle/vaccine strain	Ala	+	[35]
Gladysdale	Australia	Unknown	Cattle	Ala	+	AM114906
DVZ	Australia	1965	Cattle	Ala	+	AM114907

^a ^Strains were obtained from National Collection of Type Cultures (NCTC), PHLS, London, United Kingdom; Laboratório Nacional de Investigação Veterinária, Lisbon, Portugal; Institute of Veterinary Bacteriology, Vetsuisse Faculty, University of Bern, Bern, Switzerland; CIRAD-EMVT, Montpellier, France; and Australian Animal Health Laboratory, Geelong, Victoria, Australia.

^b ^Residue at amino acid position 204 of Bgl.

^c ^pNPbG hydrolysis within 1 h (yellow colonies also in the last of the six 10-fold dilutions) corresponds to Bgl activity and is represented by "+"; "-" indicates a reduced enzymatic activity of Bgl (only the areas spotted with the first two 10-fold dilutions became yellowish, and only after 3 h); ND: not done.

^d ^Sequences of *bgl *for PG1, Afadé and T1/Sr50 were not determined in this work, as that of PG1 is already available from the genome project [10] and those of Afadé and T1/Sr50 from our previous work [35].

**Table 2:** Oligonucleotide primers used in this study

Primer	Sequence (5'-3')^a^	Position^a^	Annealing temp. (°C)^b^	Use^c^
4000bp-6L	TCTATATCTAATCCTGAGTTTTC	954152–954174	50	P
4000bp-8R	GAACAAGGTTCAAATTGTTTTGG	954802-954780	52	S
4000bp-4L	CTAAATTGTCCTTTTATAACTGC	955230–955252	51	S
4000bp-5R	TAAACCTTACTCCTACAATACC	955347-955326	50	S
4000bp-1L	ACCATCAACTAAAACTACAGG	955499–955519	50	S
4000bp-3R	TAGAAAATATTGGTGGTTGAAC	955635-955614	49	S
4000bp-7L	TTAATCTTGTTCATTGAATAGAAG	955765–955788	51	S
4000bp-2R	CAGAAAATGATAGTGCAAATG	957029-957009	50	P
lppQTM2-L	CTAGAACTGAGGTTTTAGTAATTGGTTATGA	1166317–1166347 1190074–1190104	59	T
lppQTM2-R	CACGCTCTAGACTAATAATTTCTTCTGGTA	1166433-1166404 1190190-1190161	61	T
lppQTM2-MGB	AAAAATTTCTGGGTTTGCTCAA	1166357–1166378 1190114–1190135	53	TP

^a ^Based on nucleotide sequence NC_005364, the complete genome of *M. mycoides *subsp. *mycoides *SC type strain PG1 [10]. The *bgl *gene spans the reverse of nt 954623-956059; the two copies of *lppQ *in PG1 span nt 1165902–1167239 and nt 1189659–1190996. Note that *lppQ *occurs only in one copy in all other *M. mycoides *subsp. *mycoides *SC strains (not shown).

^b ^Obtained with the "Oligonucleotide Properties Calculator" at http://www.basic.northwestern.edu/biotools/oligocalc.html, using the nearest neighbor method and the parameters 300 nM primer and 50 mM salt (Na^+^).

^c ^P, amplification of *bgl *by PCR; S, sequencing; T, TaqMan assay for quantitative detection; TP, TaqMan probe.

**Table 3:** Viability of *M. mycoides* subsp. *mycoides* SC cell suspensions upon starvation at 37°C in medium-free buffers supplemented with 5 μM sucrose or 5 mM glucose

incubation time (h)	CFU^a^		
	
	HEPES	glucose	sucrose
strain 8740			

0	≈ 10^7^	≈ 10^7^	≈ 10^7^
4	61 × 10^5 ^(61%)	82 × 10^5 ^(82%)	93 × 10^5 ^(93%)
18	165 × 10^2 ^(0.16%)	21 × 10^3 ^(0.21%)	123 × 10^4 ^(12.3%)

strain T1/44			

0	≈ 10^7^	≈ 10^7^	≈ 10^7^
4	77 × 10^5 ^(77%)	87 × 10^5 ^(87%)	83 × 10^5 ^(83%)
18	131 × 10^2 ^(0.13%)	35 × 10^3 ^(0.35%)	104 × 10^2 ^(0.10%)

^a ^For all assays, the original CFU (at time zero) was considered as being ≈ 10^7 ^(10 μl of a suspension containing ≈ 10^9 ^CFU/ml). CFUs are reported as number of colonies formed in a specific sector of the plate X dilution factor. Numbers in parentheses represent the percentages in relation to the initial 10^7 ^CFU.

**Table 4:** Estimated growth of *M. mycoides *subsp. *mycoides *SC after treatment with 5 μM sucrose or 5 mM glucose

Pre-treatment of 18 h^a^	Ct	Estimated quantity (geq)^b^	Culture titre (mycoplasmas/ml)^c^	Growth efficacy (%)^d^
strain 8740				

none	18.26	6.94 × 10^6^	2.77 × 10^9^	100.00
HEPES	25.50	6.12 × 10^4^	2.45 × 10^7^	-0.20
glucose	22.85	3.46 × 10^5^	1.38 × 10^8^	3.94
sucrose	21.07	1.11 × 10^6^	4.42 × 10^8^	15.02

strain T1/44				

none	18.13	7.55 × 10^6^	3.02 × 10^9^	100.00
HEPES	24.85	9.35 × 10^4^	3.74 × 10^7^	0.25
glucose	23.57	2.16 × 10^5^	8.63 × 10^7^	1.88
sucrose	24.20	1.43 × 10^5^	5.72 × 10^7^	0.91

^a ^After pre-treatment with the indicated compound, mycoplasmas were grown for 2 days at 37°C in mycoplasma medium. Compounds: none, mycoplasmas were not subjected to any pre-treatment but were immediately diluted with mycoplasma medium for cultivation of 2 days at 37°C; sucrose/glucose, mycoplasmas were pre-treated for 18 h at 37°C with sucrose or glucose in incubation buffer prior to cultivation for 2 days in mycoplasma medium; HEPES, mycoplasmas were pre-treated with incubation buffer alone.

^b ^As determined by the formula geq = 1.05 × 10^12 ^× e^-0.65 × Ct ^generated by the TaqMan standard curve.

^c ^The culture titre for each test was obtained by multiplying the corresponding geq (amount of total mycoplasmas in 2.5 μl of lysate) by a factor of 400.

^d ^Calculated by first subtracting the 3 × 10^7 ^CFU of departure from each culture titre and considering then as 100% the resulting amount of non-treated mycoplasmas grown in 2-day cultures.
