# Supplementary figures and images for: β-D-Glucoside utilization by Mycoplasma mycoides subsp. mycoides SC: possible involvement in the control of cytotoxicity towards bovine lung cells
Source: BMC Microbiol. 2007 Apr 17;7:31. doi: 10.1186/1471-2180-7-31 (PMC1855930; doi:10.1186/1471-2180-7-31)

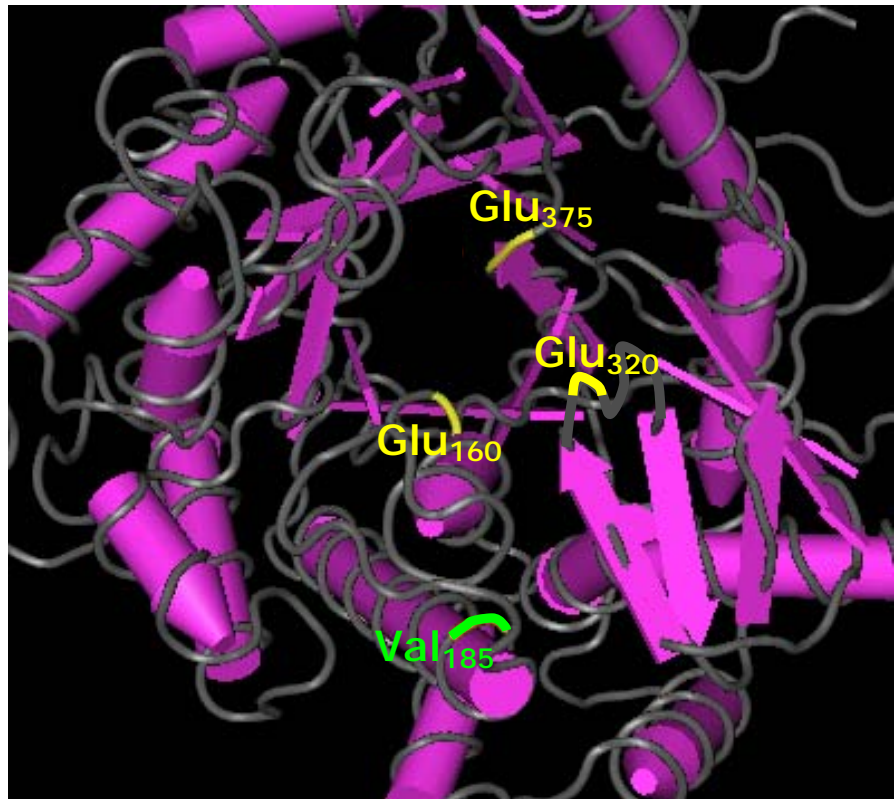

Supplement: Additional file 2 — Three-dimensional model showing the secondary structures in the vicinity of the active site of 1PBG_A. The "worm" model was obtained with the program Cn3D 4.1. The residues Glu160, Glu320 and Glu375 that comprise the catalytic site are shown in yellow. The Val185 (corresponding to Val204 in the Bgl of M. mycoides subsp. mycoides SC strain Afadé) is shown in green. All other residues are shown in pink. Note that Glu320 was not displayed by Cn3D 4.1, as Bgl amino acids 315–324 do not have a well-defined secondary structure, therefore its approximate location was introduced manually. [file 1471-2180-7-31-S2.pdf]
