# Supplementary material for: β-D-Glucoside utilization by Mycoplasma mycoides subsp. mycoides SC: possible involvement in the control of cytotoxicity towards bovine lung cells
Source: BMC Microbiol. 2007 Apr 17;7:31. doi: 10.1186/1471-2180-7-31 (PMC1855930; doi:10.1186/1471-2180-7-31)
Supplement: Additional file 1 — Alignment of the Bgl amino acid sequence with those of related proteins. The Bgl sequence from strain Afadé was aligned with the protein sequences COG2723, pfam00232, 1PBG_A, 1HXJ_A and 1GOW_A using the program DIALIGN 2, and the alignment was elaborated with Boxshade 3.21. The sequences for COG2723 (from Enterococcus faecium) and pfam00232 (from Streptococcus pneumoniae) were obtained with blastp and showed very high similarities with the Bgl sequence from M. mycoides subsp. mycoides SC. Black backgrounds indicate identical amino acids and grey backgrounds indicate similar residues. Asterisks (*) indicate the three Glu residues of the catalytic site and the circle (o) indicates the amino acid 204 in the Bgl sequence from M. mycoides subsp. mycoides SC. [file 1471-2180-7-31-S1.pdf]

```
Bgl          1 MMLIFMELIFYIKRIYMFKFKKDFWWGAASSGCQTESDKDKPN--LNIMDYWYKQTPTDF
COG2723      1 M-KK-------------YQFPANFWWGSAASGPQTEGRISGDGKGENIFDYWYKKEPQKF
pfam00232    1 MKKK-------------LVFPNLFWWGAASSGPQTEGQYGKVH--ENVMDYWFKTHPEDF
1PBG-A       1 MTK---------------TLPKDFIFGGATAAYQAEGATHTDGKGPVAWDKYLEDNYWYT
1HXJ-A       1 SQNGVQMLSPSEIPQRDW-FPSDFTFGAATSAYQIEGAWNEDGKGESNWDHFCHNHPERI
1GOW-A       1 ----------------MYSFPNSFRFGWSQAGFQSEMGTPGSEDPNTDWYKWVHDPENMA

Bgl         59 YD---NKGPNITCDTYSNYKTDVKLMSEIGLNSFRTSIQWTRLIKNLYT--GE-------
COG2723     47 FD---QVGPEKTSQVYTKYQEDVQLMKQTGHNSFRTSIQWSRLIPD-ES--GT-------
pfam00232   46 FD---NVGPLVASNFFHTYTEDFHLMKEIGVNSFRTSIQWSRLIKNLET--GE-------
1PBG-A      46 A--------EPASDFYHKYPVDLELAEEYGVNGIRISIAWSRIFPT-GY--GE-------
1HXJ-A      60 LD---GSNSDIGANSYHMYKTDVRLLKEMGMDAYRFSISWPRILPK-GTKEGG-------
1GOW-A      45 AGLVSGDLPENGPGYWGNYKTFHDNAQKMGLKIARLNSEWSRQFPNPLPRPQNFDESKQD

Bgl        107 -----------------VDLKQVEFYRNYFLEIKKNNIKLIVNLFHFDTPIELENI----
COG2723     94 -----------------INEEAVRFYHRYFDELIENGIEPFVNLYHFDMPMYLQEK----
pfam00232   94 -----------------PDPKGIAFYNAIIEEAKKNQMDLVMNLHHFDLPVELLQKY---
1PBG-A      88 -----------------VNEKGVEFYHKLFAECHKRHVEPFVTLHHFDTPEALHSN----
1HXJ-A     109 -----------------INPDGIKYYRNLINLLLENGIEPYVTIFHWDVPQALEEKY---
1GOW-A     105 VTEVEINENELKRLDEYANKDALNHYREIFKDLKSRGLYFIQNMYHWPLPLWLHDPIRVR

                                                           *
Bgl        146 -------GGW---TNKKTVELYFLYAKQCFKYFSDLVDYWTTFNEPVVLVDGCYLNKW--
COG2723    133 -------GGW---LNRETVEAYEKYAKSCFELFGSKVKKWFTHNEPIVPVEAGYLYRW--
pfam00232  134 -------GGW---ESKHVVELFVKFAKTAFTCFGDKVHYWTTFNEPMVIPEAGYLYAF--
1PBG-A     127 -------GDF---LNRENIEHFIDYAAFCFEEFP-EVNYWTTFNEIGPIGDGQYLVGK--
1HXJ-A     149 -------GGFLDKSHKSIVEDYTYFAKVCFDNFGDKVKNWLTFNEPQTFTSFSYGTGV--
1GOW-A     165 RGDFTGPSGW---LSTRTVYEFARFSAYTAWKFDDLVDEYSTMNEPNVVGGLGYVGVKSG

                                     o
Bgl        194 YYPKI------------SNLKLVVQAAYNTILAHCKVANYFHSYFKNDQ--NKKISIILN
COG2723    181 HYPEE------------SDMKKAIQVGYHEALASALAIK---SYHEMEQ--GGVIGIILN
pfam00232  182 HYPNLKGKG-----------KEAVQVIYNLNLASAKVIQ---LYRSLEL--DGKIGIILN
1PBG-A     174 FPPGIKY-----------DLAKVFQSHHNMMVSHARAVK---LYKDKGY--KGEIGVVHA
1HXJ-A     200 FAPGRCSPGLDCAYPTGNSLVEPYTAGHNILLAHAEAVD---LYNKHYKRDDTRIGLAFD
1GOW-A     222 FPPGY------------LSFELSRRAMYNIIQAHARAYDGIKSVSKKP------VGIIYA

Bgl        240 LTPTIPKN-SELKHLKAAKIRDELLNKSFLNAVIKGQFSTFLIKFLN--------ENNLM
COG2723    224 LTPSYPRDESNPEDVKAAQIADAFFNRSFLDPAVKGEFPSELIEIVK--------ELDLM
pfam00232  226 LTPAYPRS-NSPEDLEASRFTDDFFNKVFLNPAVKGTFPERLVKQL--------------
1PBG-A     218 LPTKYPYDPENPADVRAAELEDIIHNKFILDATYLGHYSDKTMEGVNHILAENGGELDLR
1HXJ-A     257 VMGRVPYG-TSFLDKQAEERSWDINLGWFLEPVVRGDYPFSMRSLAR--------ERLPF
1GOW-A     264 NSS---FQPLTDKDMEAVEMAENDNRWWFFDAIIRGEITRGNEKIV--------------

                                                                      *
Bgl        291 PEYDQL-----------ELNEIKKTRIDFLAVNYYQPARVQAPLNNLTSSTELKLENWFL
COG2723    276 PVIHA-----------EDLTVIKENTVDLLGINYYQPRRVKAKETPIDTKNGPMPEDYFD
pfam00232  271 -----ERDGVLWSHTEKELQLMKSNTVDFLGVNYYHPKRVQAQANPEEYQTPWMPDQYFK
1PBG-A     278 DEDFQALDAAKDLN-------------DFLGINYYMSDWMQAFDGETEIIHNGKGEKGSS
1HXJ-A     308 FKDEQKEKL--------------AGSYNMLGLNYYTSRFSKNID----------------
1GOW-A     307 -----RDDL--------------KGRLDWIGVNYYTRTVVKRTEKGYVSLGGYGHGCERN

Bgl        340 PYTNKNIRINPYR--------GWE------------------------IHPQTLYDIAID
COG2723    325 NYEMPGRKMNPYR--------GWE------------------------IYEQGIYDILIN
pfam00232  326 EYEWLERRMNPYR--------GWE------------------------IFPKAIYDIAMI
1PBG-A     325 KYQIKGVGRRVAPDYVPRTDWDWI------------------------IYPEGLYDQIMR
1HXJ-A     338 ----------ISPNYSPVLNTDDAYASQEVNGPDGKPIGPPMGNPWIYMYPEGLKDLLMI
1GOW-A     348 SVSLAG---------LPTSDFGWE------------------------FFPEGLYDVLTK

                             *
Bgl        368 IKNNYDNIP-WIVSENGIGVSDENRF-LNKQGYIDDQYRIDFIKEHLIYLYKAIEQGSNC
COG2723    353 VRDHYGNID-CFISENGMGVENEARF-IKEDGMIHDDYRIEFVQSHLKYVHQAIQEGANC
pfam00232  354 VKEEYGNIP-WFISENGMGVENEARF-IDENGVIDDVYRIEFYEEHLRWLHKAIEEGSHC
1PBG-A     361 VKNDYPNYKKIYITENGLGYKDEFV-----DNTVYDDGRIDYVKQHLEVLSDAIADGANV
1HXJ-A     388 MKNKYGNPP-IYITENGIGDVDTKETPLPMEAALNDYKRLDYIQRHIATLKESIDLGSNV
1GOW-A     375 YWNRYHLY--MYVTENGIADDA-------------DYQRPYYLVSHVYQVHRAINSGADV

Bgl        426 FGYQMWTLIDNWSWANGFKNRYGFISLDTKTL-KRTIKKSGYWIKQVIKDQGFE------
COG2723    411 LGYHMWTCMDNWSWTNAYKNRYGFISVDLAQDGKRTIKKSGHWFKDVSDKNGFEA-----
pfam00232  412 FGYHAWTAFDCWSWNNAYKNRYGFISVDLETQ-KRTIKSSGRWYRKVSDNNGFEVEIEE-
1PBG-A     416 KGYFIWSLMDVFSWSNGYEKRYGLFYVDFDTQ-ERYPKKSAHWYKKLAETQVIE------
1HXJ-A     447 QGYFAWSLLDNFEWFAGFTERYGIVYVDRNNNCTRYMKESAKWLKEFNTAKKPSKKILTP
1GOW-A     420 RGYLHWSLADNYEWASGFSMRFGLLKVDYNTK-RLYWRPSALVYREIATNGAITDEIEHL

Bgl            -----------
COG2723        -----------
pfam00232      -----------
1PBG-A         -----------
1HXJ-A     507 A----------
1GOW-A     479 NSVPPVKPLRH
```
